# Identification and Functional Studies on the Role of *PlSPL14* in Herbaceous Peony Stem Development

**DOI:** 10.3390/ijms25158443

**Published:** 2024-08-02

**Authors:** Huajie Xu, Renkui Yu, Yuhan Tang, Jiasong Meng, Jun Tao

**Affiliations:** 1 College of Horticulture and Landscape Architecture, Yangzhou University, Yangzhou 225009, China; 2 Joint International Research Laboratory of Agriculture and Agri-Product Safety, The Ministry of Education of China, Yangzhou University, Yangzhou 225009, China

**Keywords:** *Paeonia lactiflora*, *PlSPL14*, xylem development, functional verification, yeast two-hybrid assay

## Abstract

Stem strength plays a crucial role in the growth and development of plants, as well as in their flowering and fruiting. It not only impacts the lodging resistance of crops, but also influences the ornamental value of ornamental plants. Stem development is closely linked to stem strength; however, the roles of the SPL transcription factors in the stem development of herbaceous peony (*Paeonia lactiflora* Pall.) are not yet fully elucidated. In this study, we obtained and cloned the full-length sequence of *PlSPL14*, encoding 1085 amino acids. Quantitative real-time PCR (qRT-PCR) analysis revealed that the expression level of *PlSPL14* gradually increased with the stem development of *P. lactiflora* and was significantly expressed in vascular bundles. Subsequently, utilizing the techniques of virus-induced gene silencing (VIGS) and heterologous overexpression in tobacco (*Nicotiana tabacum* L.), it was determined that *PlSPL14*-silenced *P. lactiflora* had a thinner xylem thickness, a decreased stem diameter, and weakened stem strength, while *PlSPL14*-overexpressing tobacco resulted in a thicker xylem thickness, an increased stem diameter, and enhanced stem strength. Further screening of the interacting proteins of PlSPL14 using a yeast two-hybrid (Y2H) assay revealed an interactive relationship between PlSPL14 and PlSLR1 protein, which acts as a negative regulator of gibberellin (GA). Additionally, the expression level of *PlSLR1* gradually decreased during the stem development of *P. lactiflora*. The above results suggest that *PlSPL14* may play a positive regulatory role in stem development and act in the xylem, making it a potential candidate gene for enhancing stem straightness in plants.

## 1. Introduction

The formation and development of stems are crucial for the growth of ornamental plants, which will further affect the ornamental value and cut flower quality [1]. Stems play important roles in conduction, support, and storage, with the support function required to bear the weight of all above-ground organs and withstand adverse weather conditions [2]. Herbaceous peony (*Paeonia lactiflora* Pall.), is renowned for its large flowers, which necessitate sturdy upright stems to ensure optimal ornamental value. Therefore, insufficient stem strength will significantly compromise the quality of cut flowers and diminish their commercial value. Stem bending affects the uptake of water and other nutrients by the plant, and stem breaking also disrupts the stem transport system, affecting the movement of photosynthesis products from leaves to grains and leading to plant mortality and yield loss in adverse cases [3]. Stem strength is essential for stem development as it influences the degree of stem straightness. Plant morphology, including height and stem diameter, along with anatomical characteristics, like conducting and mechanical tissues, and vascular tissues (xylem, cambium, and phloem), all impact plant stem strength [4].

The xylem, consisting mainly of tracheary elements such as vessels and fibers with highly thickened secondary cell walls (SCWs) [5], serves primarily for the conduction of water and nutrients as well as providing mechanical support in vascular tissue [6]. The mechanical support strength of xylem is determined by the number of xylem cell layers and xylem thickness [7], which is closely associated with the proliferation and differentiation of cells in the vascular cambium layer during xylem development [8]. The cells in the vascular cambium layer of plant stems differentiate outwardly into phloem and inwardly into xylem [9,10], primarily responsible for transporting water and solute minerals [11]. The xylem is categorized into primary and secondary xylem, and the development of secondary xylem is a dynamic process that begins with cell proliferation in the vascular cambium and progresses through cell expansion and differentiation, secondary cell wall (SCW) thickening, and programmed cell death [12]. In recent years, extensive research on xylem development has revealed that phytohormones, transcription factors, and polypeptide signals play crucial roles in this process [13,14,15]. Among them, transcription factors are recognized as critical regulators of xylem development; many studies have indicated their involvement in regulating xylem development [16]. For instance, in *Populus trichocarpa* Torr.&Gray, *PtrHB4* is part of the HD-ZIP Ⅲ family; the repression of *PtrHB4* caused defects in the formation of the interfascicular cambium, and the overexpression of *PtrHB4* induced cambium activity and xylem differentiation [17]. MYB transcription factors mainly affect xylem differentiation by regulating secondary cell wall thickening mechanisms. In *P. lactiflora*, multiple R2R3-MYB transcription factors are specifically expressed in stems, regulating stem strength to enhance plant lodging resistance by regulating secondary cell wall thickening [18]. Recently, NAC transcription factors were found to play an important regulatory role in xylem development. In *Populus deltoides* Marshall, the overexpression of *PdWND3A*, a wood-associated NAC domain-containing protein, increases xylem vessels and lignin content in the stem [19]. In summary, xylem development is regulated by complex transcription factors, which in turn affect the stem development of plants.

SPL (Squamosa-promoter Binding Protein, SBP) is a plant-specific transcription factor that is widely found in green plants. SPL transcription factors have been broadly characterized in a variety of plants, including *Arabidopsis thaliana* L. [20], *Oryza sativa* L. [21], *Brassica campestris* L. [22], and *Vitis vinifera* L. [23]. Further research on its function found that SPL transcription factors can regulate plant cold tolerance, drought resistance, flowering, plant architecture, tillering, and ear structure, etc. In *A. thaliana*, *SPL9* activates the expression of the downstream target gene *CBF2*, improving the freezing tolerance of Arabidopsis [20]; *SPL3* is regulated by microRNA to control the formation of flowering [24], and *microRNA156*-targeted *SPL9* can control the quantity and distribution of trichomes in Arabidopsis [25]. *OsSPL10* from *O. sativa* responds to drought tolerance by controlling reactive oxygen species (ROS) production and stomatal movement [26]. In *Triticum aestivum* L., *TaSPL13* improves wheat yield by increasing the size and quantity of grain and improving plant structure [27]. Although there have been many reports on the function of *SPL*, only a few *SPLs* have been reported to regulate stem development, *O. sativa SPL14* (*OsSPL14*), identified as the IDEAL PLANT ARCHITECTURE1 or WEALTHY FARMER’S PANICLE gene, is critical in regulating rice plant architecture. The overexpression of *OsSPL14* plants showed an increase in stem diameter and stem straightness, promoting stem development [28,29]. Similarly, *OsSPL14* can affect the grain yield of rice by affecting the stem strength, tiller number, and ear branch number [30]. However, their regulatory mechanisms have not been thoroughly investigated, let alone in *P. lactiflora* [31]. Recently, *PlSPL1* from *P. lactiflora* was reported for the first time as possibly playing an important role in the degree of stem straightness [32], but the specific function has not been verified. Therefore, it is of great significance to identify SPLs related to stem development and explore their potential regulatory mechanisms that promote xylem thickness and stem strength.

In this study, we cloned an SPL transcription factor, PlSPL14, and then characterized it using bioinformatics methods. Subsequently, the expression characterization of *PlSPl14* was analyzed using qRT-PCR and subcellular localization. Furthermore, the function of *PlSPL14* in stem development was further investigated through virus-induced gene silencing (VIGS) and heterologous overexpression. In addition, its interacting protein was further confirmed using a yeast two-hybrid (Y2H) assay, which will provide the groundwork for further study into the mechanism of *PlSPL14* in regulating the stem development of *P. lactiflora*.

## 2. Results

### 2.1. Identification and Phylogenetic Analysis of PlSPL14

**Figure 1:**
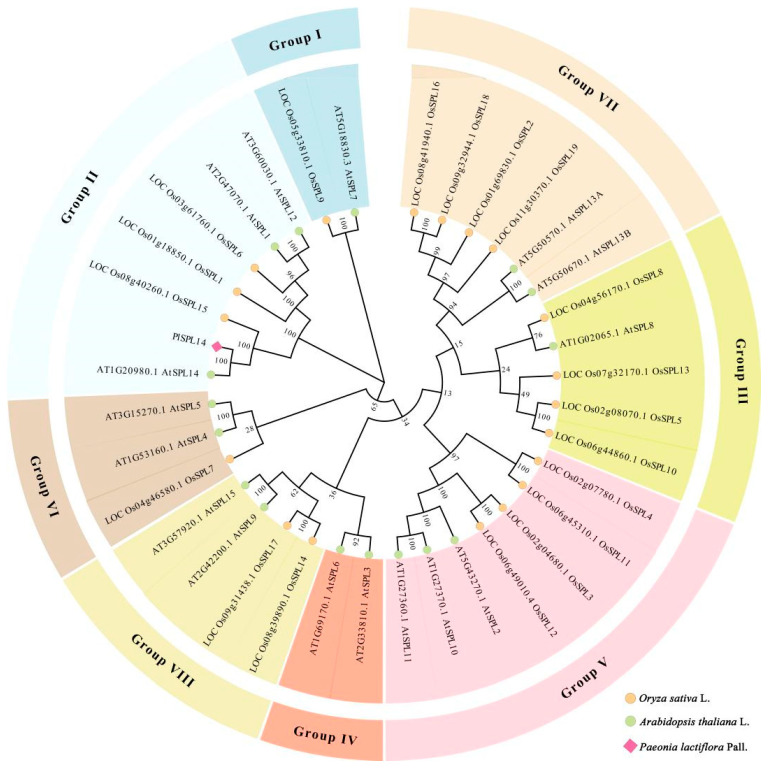
Phylogenetic analysis of SPL proteins from herbaceous peony (*P. lactiflora*), arabidopsis (*A. thaliana*), and rice (*O. sativa*). Different colors represent different subfamilies, with genes that have closer evolutionary relationships clustering together to form subgroups. The neighbor-joining (NJ) method was adopted, and the bootstrap value was set to 1000. At: *Arabidopsis thaliana* L.; Os: *Oryza sativa* L.

SPL14-like transcription factors in plants have been demonstrated to be associated with stem development [30,33]. Based on the transcriptome database, the full-length sequence of *PlSPL14* (GeneBank accession number: PP861171) in *P. lactiflora* was identified and isolated using polymerase chain reaction (PCR) technology. The full-length sequence of *PlSPL14* was determined to be 4020 bp and presented a 232-bp 5′ untranslated region (UTR) and a 530-bp 3′ UTR, with a complete open reading frame (ORF) of 3258 bp, encoding 1085 amino acids. The physicochemical analysis of the SPL protein revealed a molecular weight of 11,976 kDa, a theoretical isoelectric point of 8.68, and an instability index of 52.17. A phylogenetic analysis was performed on the PlSPL14 protein with sequences of 16 SPLs from *A. thaliana* and 19 SPLs from *O. sativa*, which were classified into a total of 8 subfamilies, including Group I, Group II, Group III, Group IV, Group V, Group VI, Group VII, and Group VIII, of which PlSPL14 belonged to Group II (Figure 1). It is evident that PlSPL14 is closely related to AtSPL14 and forms a cluster with it. 

**Figure 2:**
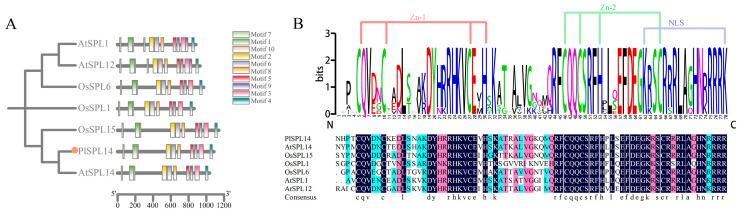
Multiple sequence alignments for the phylogenetic tree and the conserved domains of the Group II subfamily. (**A**) Motif compositions of the Group II subfamily. A total of 10 motifs are shown as rectangles with different colors. (**B**) Predicted SBP domain of Group II subfamily members and its sequences logos. The letters represent amino acids, larger colored letters indicate a higher level of similarity at that position among the proteins.

PlSPL14 protein showed sequence alignment with Arabidopsis thaliana AtSPL1, AtSPL12, AtSPL14, and Oryza sativa OsSPL1, OsSPL6, and OsSPL15 proteins, all of which belong to the Group II subfamily. Subsequently, we conducted an analysis of their conserved motifs using the MEME server. They all contain 10 conserved motifs, and the distribution of motifs is roughly the same, which proves that the gene structure within the subfamily is similar (Figure 2A). As shown in Figure 2B, PlSPL14 possesses an SBP-conserved structural domain typical of the SPL transcription factor family [34] for binding to DNA sequences. They share the same SBP structural domain consisting of approximately 78 amino acids, which includes two zinc finger structural domains—the first one is at the N-terminal end, and Zn-1 has two types, C3H (Cys-Cys-Cys-His) or C4 (Cys-Cys-Cys-Cys-Cys, Cys4), while the second one is at the C-terminal end, and Zn-2 is generally of the C2HC (Cys-Cys-His-Cys) type—as well as a nuclear localization signal (NLS) (KR/C-RRRK/R). 

### 2.2. Expression and Characteristic Analysis of PlSPL14

**Figure 3:**
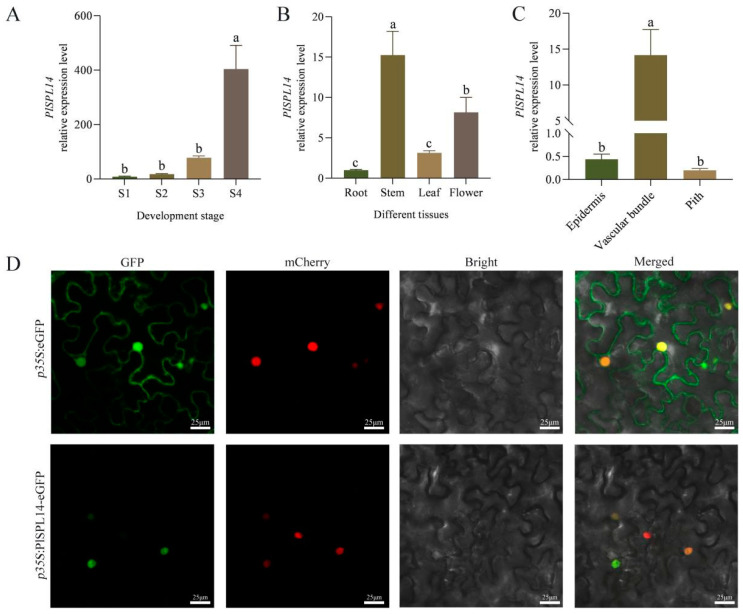
Expression properties of *PlSPL14*. (**A**) Temporal expression patterns of *PlSPL14* at four different developmental periods (S1–S4). Data are means ± SDs, and letters indicate significant differences (*p* < 0.05). (**B**) Spatial expression patterns of *PlSPL14*. (**C**) Expression pattern of *PlSPL14* in different tissues of the stem. (**D**) Subcellular localization of PlSPL14-eGFP fusion protein in *N. benthamiana* leaf epidermal cells. The mCherry protein indicates nucleus localization. S1, flower-bud stage, S2, pigmented stage, S3, unfolded-petal stage, and S4, full-bloom stage.

To verify the expression trend of *PlSPL14* with the period of stem development, we performed qRT-PCR at the indicated time points. Our results showed that the expression of *PlSPL14* increased continuously from S1 to S4 with the stem development of *P. lactiflora* reaching a peak at S4 with a 50-fold increase (Figure 3A), consistent with the transcriptome database [35]. Additionally, we examined *PlSPL14* expression in different tissues such as roots, stems, leaves, and flowers, and found that *PlSPL14* expression in stems was higher than in other tissues during the S4 period (Figure 3B). In order to determine the specific regions of the stem with higher expression, we isolated distinct tissues, including the epidermis and cortex, vascular bundles, and pith. Our findings revealed an elevated expression of *PlSPL14* in the vascular bundles compared to the other two tissues (Figure 3C).

The subcellular localization of PlSPL14 was determined by fluorescence imaging (Figure 3D). The control pCAMBIA2300-eGFP construct exhibited a uniform distribution of green fluorescence throughout the cell, while the signals from the pCAMBIA2300-PlSPL14-eGFP fusion protein were colocalized with the NLS marker signals, indicating a nuclear localization of PlSPL14.

### 2.3. Silencing PlSPL14 in P. lactiflora Negatively Regulates Stem Development by Attenuating Stem Strength

**Figure 4:**
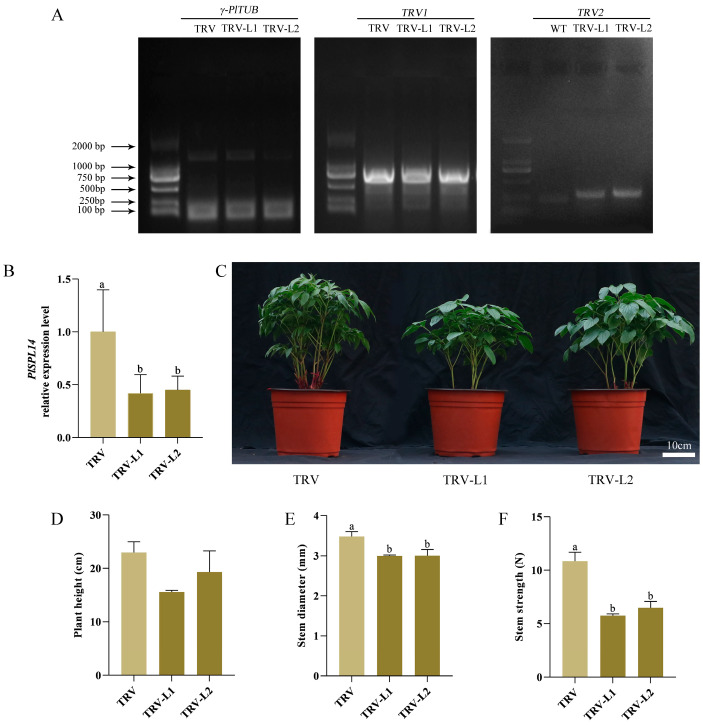
Identification and phenotypic observation of *PlSPL14*-silenced *P. lactiflora*. (**A**) PCR validation of *PlSPL14*. (**B**) qRT-PCR analysis of *PlSPL14*. (**C**) Phenotype of TRV and silencing lines. (**D**) Plant height. (**E**) Stem diameter. (**F**) Stem strength. Data are means ± SDs, and letters indicate significant differences (*p* < 0.05) by the Student’s *t*-test. Bars in (**D**–**F**) are means ± SDs of *n* = 3 biological replicates. Error bars represent standard deviations of three biological replicates.

**Figure 5:**
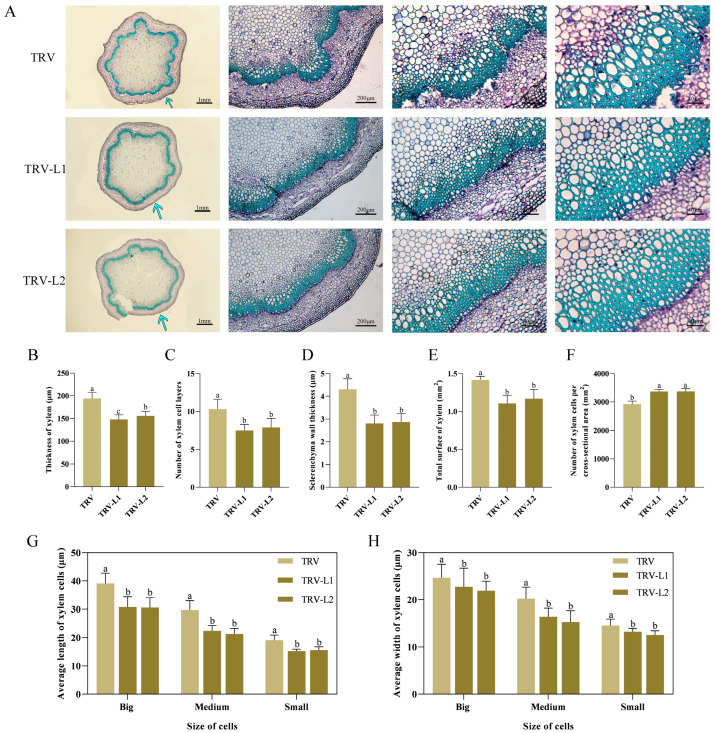
Stem microstructures observation in TRV and silencing lines. (**A**) Stem microstructures observation under optical microscopes after 0.05% tolonium chloride solution staining. Representative images of *n* = 3 replicates per line are shown. The arrows indicate the areas observed in the second anatomical image on the right. (**B**) Thickness of xylem. (**C**) Number of xylem cell layers. (**D**) Sclerenchyma wall thickness. (**E**) Total surface of xylem. (**F**) Number of xylem cells per cross-sectional area. (**G**) Average length of xylem cells. (**H**) Average width of xylem cells. Results are mean ± SDs, and letters indicate significant differences (*p* < 0.05). Data were collected from 30 measurements of three individual plants.

To explore the putative function of *PlSPL14* in enhancing stem strength in *P. lactiflora*, we transiently silenced *PlSPL14* expression using VIGS technology to obtain a *PlSPL14*-silenced plant and validated the silenced plants using PCR analysis (Figure 4A). The qRT-PCR analysis revealed a 55% reduction in *PlSPL14* expression levels in *P. lactiflora* compared to the TRV empty vector-transformed plants (Figure 4B). Phenotypic observation indicated that the *PlSPL14*-silenced *P. lactiflora* grew relatively weakly (Figure 4C). Morphological index measurements showed that plant height was slightly reduced compared with the TRV empty vector-transformed plants (Figure 4D). Additionally, the stem strength and stem diameter in the *PlSPL14*-silenced *P. lactiflora* lines were significantly lower than in the TRV empty vector-transformed plants (Figure 4E,F). Subsequently, to observe the differences in stem structure between the plants, the microstructures of the stems of *PlSPL14*-silenced *P. lactiflora* and TRV empty vector-transformed plants were stained and observed with tolonium chloride (Figure 5A). This showed that xylem development was hindered after the silencing of *PlSPL14*, resulting in a 25% reduction in xylem thickness (Figure 5B), and a reduction of 10% in the number of xylem cell layers (Figure 5C). Simultaneously, it was obvious that the sclerenchyma wall thickness in the xylem became thinner in the *PlSPL14*-silenced *P. lactiflora*, which was confirmed by the statistics; in particular, the sclerenchyma wall thickness was dramatically decreased by 34.24%. (Figure 5D). Moreover, the total surface of the xylem in the TRV empty vector-transformed plants was larger than in the *PlSPL14*-silenced *P. lactiflora* (Figure 5E); the number of xylem cells per cross-sectional area in the TRV empty vector-transformed plants was less than in the *PlSPL14*-silenced *P. lactiflora* (Figure 5F). The average length and width of xylem cells in the TRV empty vector-transformed plants were larger than in the *PlSPL14*-silenced *P. lactiflora* (Figure 5G,H). 

### 2.4. PlSPL14-Overexpressing Plant Exhibits Increased Stem Strength

**Figure 6:**
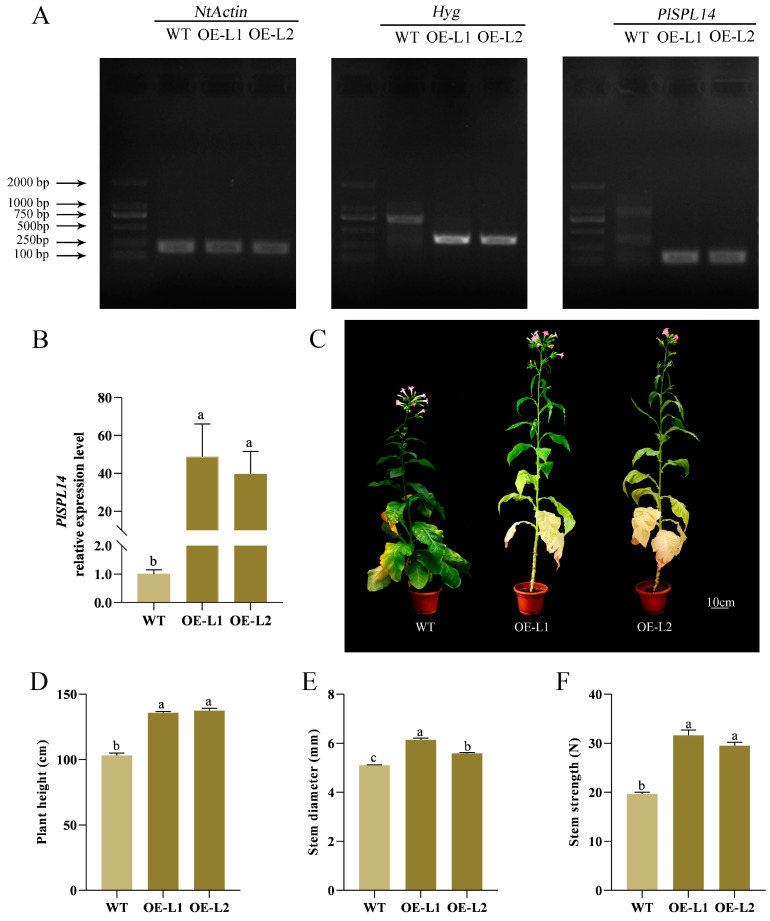
Identification and phenotypic observation of WT and *PlSPL14*-overexpressing lines. (**A**) PCR validation of *PlSPL14*. (**B**) qRT-PCR analysis of *PlSPL14*. (**C**) Phenotype of WT and *PlSPL14*-overexpressing lines. (**D**) Plant height. (**E**) Stem diameter. (**F**) Stem strength. Data are means ± SDs, and letters indicate significant differences (*p* < 0.05) by the Student’s *t*-test. Bars in (**D**–**F**) are means ± SDs of *n* = 3 biological replicates.

**Figure 7:**
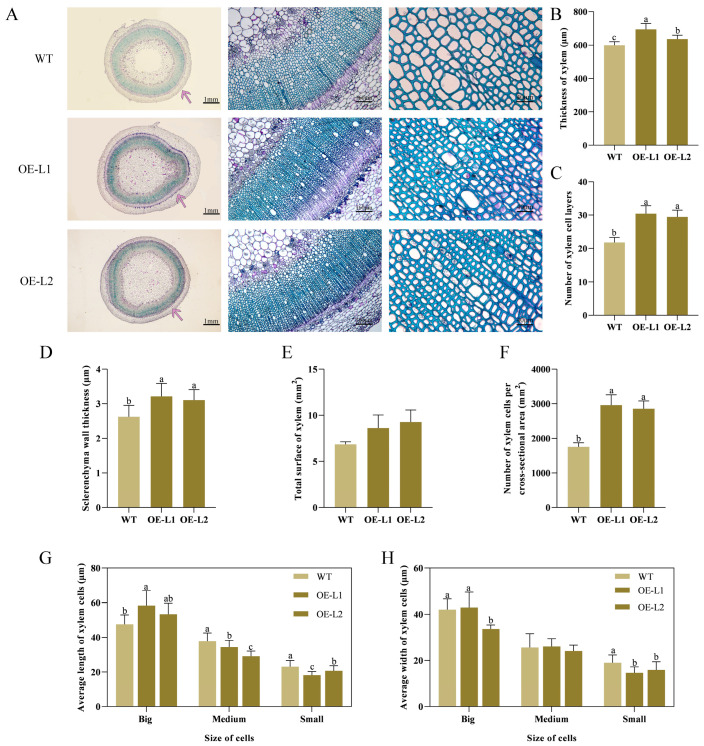
Stem microstructures observation in WT and *PlSPL14*-overexpressing lines. (**A**) Stem microstructures observation under optical microscopes after 0.05% tolonium chloride solution staining. Representative images of *n* = 3 replicates per line are shown. The arrows indicate the areas observed in the second anatomical image on the right. (**B**) Thickness of xylem. (**C**) Number of xylem cell layers. (**D**) Sclerenchyma wall thickness. (**E**) Total surface of xylem. (**F**) Number of xylem cells per cross-sectional area. (**G**) Average length of xylem cells. (**H**) Average width of xylem cells. Results are means ± SDs, and letters indicate significant differences (*p* < 0.05). Data were collected from 30 measurements of three individual plants.

To further validate the function of *PlSPL14* in regulating the stem development of *P. lactiflora*, two independent overexpressing tobacco (*N. tabacum*) lines were generated by introducing the pCAMBIA1301-*PlSPL14* vector for heterologous overexpression in tobacco (Figure 6A). The qRT-PCR analysis revealed a 40-fold increase in *PlSPL14* expression levels in the overexpressing lines compared to the wild type (WT) plants (Figure 6B). As shown in Figure 6C, the transgenic lines exhibited significantly stronger phenotypes with increased plant height, straighter stems, and stronger growth than the WT. The morphological index measurements showed that the plant height and stem strength in the *PlSPL14*-overexpressing lines were significantly higher than the WT (Figure 6D,F), while there was only a slight difference in stem diameter compared to WT (Figure 6E). Furthermore, the microstructures of the stems of the *PlSPL14*-overexpressing lines and WT plant were stained and observed with tolonium chloride (Figure 7A), and the thickness of the xylem was increased in all transgenic lines by an average of 22.44% (Figure 7B), while there was an increase in the number of xylem cell layers by 36.03% compared with the WT (Figure 7C). Simultaneously, it was obvious that the sclerenchyma wall thickness in the xylem became thicker in the *PlSPL14*-overexpressing lines, which was confirmed by the statistics; in particular, the sclerenchyma wall thickness was dramatically increased by 20.23%. (Figure 7D). Moreover, the total surface of xylem in the WT plants was smaller than in the *PlSPL14*-overexpressing lines (Figure 7E), and the number of xylem cells per cross-sectional area in the WT plants was less than in the *PlSPL14*-overexpressing lines (Figure 7F). The average length and width of xylem small cells in the WT plants were larger than in the *PlSPL14*-overexpressing lines (Figure 7G,H). 

### 2.5. Analysis of the Protein Interaction of PlSPL14

**Figure 8:**
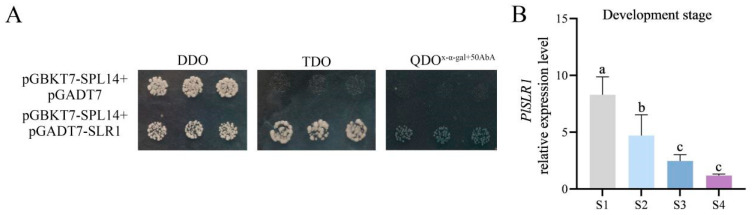
Analysis of the PlSPL14-interacting proteins. (**A**) Y2H assays to identify the interactions between PlSPL14 and PlSLR1. (**B**) Temporal expression patterns of *PlSLR1* at four different stem developmental periods (S1–4). Data are means ± SDs, and letters indicate significant differences (*p* < 0.05).

To screen for proteins interacting with PlSPL14, a yeast two-hybrid library screening system assay was used. Firstly, the self-activation activity of the pGBKT7-PlSPL14 bait gene was examined, revealing that all transformants grew on the SD medium lacking Leu and Trp (SD/-TL, DDO). At the same time, growth was effectively inhibited on an SD medium lacking Leu, Trp, and His (SD/-TLH, TDO), indicating that the yeast two-hybrid system can be used to screen interacting proteins with PlSPL14 (Figure 8A). Furthermore, after comparison and analysis, the DELLA protein SLENDER RICE 1-LIKE (designated PlSLR1) number 4 was selected for cloning and subsequent analysis. The protein PlSLR1 is a negative regulator of gibberellin signaling and belongs to the GRAS transcription factor gene family, encoding 539 amino acids. The interaction between PlSPL14 and PlSLR1 was verified again by a dot-plate test of a yeast two-hybrid assay. When transferred onto the SD medium lacking Leu, Trp, Ade, and His (SD/-TLHA, QDO) with 50 ng mL^−1^ of AbA, only the yeast containing both PlSPL14 and PlSLR1 could grow normally and turn blue, which indicated that PlSPL14 interacted with PlSLR1 in yeast (Figure 8A). In addition, the expression level of *PlSLR1* dropped dramatically with the stem development in *P. lactiflora*, and its expression trend was significantly negatively correlated with the variation trend of *PlSPL14* (Figure 8B).

## 3. Discussion

Stem development is crucial for plant growth and various biological functions. Among the factors influencing stem development, the mechanical strength of the stem is the main indicator of stem straightness [36]. Therefore, studying the molecular mechanisms that affect the stem strength of *P. lactiflora* and regulating them has important theoretical and practical significance for improving the stem straightness of *P. lactiflora* and enhancing the production level of cut flowers. In this study, we found that *P. lactiflora* could modulate the thickness of the xylem to improve stem strength through regulation via an SPL transcription factor, PlSPL14. 

SPL transcription factors contain a highly conserved SBP domain with a 78 amino acid DNA-binding domain, which includes two zinc-finger domains, Zn-1 (C3H or C4) and Zn-2 (C2HC), as well as a nuclear localization signal (KR/C-RRRK/R) [34]. At present, SPLs have been widely identified in plants. For example, Peng [37] identified *EjSPL14* from loquat (*Eriobotrya japonica* (Thunb.) Lindl.), which encodes a protein containing 1076 amino acids and possesses a conserved SPB domain. Liang et al. [38] identified *AtSPL14* from Arabidopsis (*A. thaliana*), which encodes a protein containing 1035 amino acids with an SBP DNA-binding domain. Recently, another gene belonging to the SPL family, *PlSPL1*, was reported in the herbaceous peony [32]. In addition, we conducted a comparative analysis of the key domains, phylogenetic relationship, and physicochemical property analysis between PlSPL1 and PlSPL14. Our findings revealed that both proteins belong to the SPL transcription factor family (Figure S1). Phylogenetic relationship analysis demonstrates that they cluster together on the same evolutionary branch, specifically within the Group II subfamily (Figure S2). However, an examination of their physicochemical properties indicates distinct differences between the two proteins (Figure S3). *PlSPL1*, encoding 999 amino acids, harbors a conserved SBP domain and shares homology with *AtSPL1*. Notably, the expression level of *PlSPL1* gradually decreased during stem development, contrasting with the expression pattern of *PlSPL14* reported herein. This suggests potential functional disparities between them, and further investigation into the specific role of *PlSPL1* is warranted. In this study, the full-length *PlSPL14* cDNA was obtained using RACE technology, which encodes a protein of 1085 amino acids and contains Zn-1, Zn-2, and NLS domains, confirming that this sequence is an SPL transcription factor. Additionally, *PlSPL14* was found to belong to Group II in terms of phylogenetic relationships (Figure 1), which is homologous to *A. thaliana SPL14*. The expression of SPL is distributed differently in different species and has tissue specificity. *SPL3* and *SPL5* from *A. thaliana*, which could regulate the flowering time, showed the highest expression level at the shoot apical meristem (SAM) [39]. In *Brassica juncea* (L.) Czern., *BjuSPL3a-B*, *BjuSPL2b-B,* and *BjuSPL2c-A* were significantly expressed in flowers and may perform functional roles there, while *BjuSPL3b-B* and *BjuSPL10a-A* were significantly expressed in stems to promote stem development [40]. A similar expression pattern was observed in this study, indicating that *PlSPL14* exhibited high expression levels in stems, especially in the vascular bundles. These results implied that *PlSPL14* might be positively involved in regulating the stem development of *P. lactiflora*.

Previous studies have shown that SPL has been reported to have multiple functions. *O. sativa SPL14*, also known as the Ideal Plant Architecture 1 (IPA1), promotes grain yield and blast resistance by facilitating the expression of downstream transcription factor genes [21,28,29]. In *Medicago sativa* L., *SPL9* RNAi plants exhibit a reduced stem diameter and plant height and promote drought tolerance in alfalfa, possibly by regulating anthocyanin biosynthesis, when subjected to abiotic stress drought [41]. By interfering with starch metabolism via the gibberellin (GA) pathway, *O. sativa IPA1* adversely affects the germination of rice seeds and the growth of young seedlings [42]. In this study, the gene function of *PlSPL14* was first identified. The function of *PlSPL14* was first investigated using VIGS technology. The growth of *PlSPL14*-silenced *P. lactiflora* was comparatively feeble (Figure 4C), and stem strength was decreased (Figure 4F). Moreover, the xylem thickness and xylem cell layer number were decreased (Figure 5B,C). When compared with the TRV empty vector-transformed plants, the *PlSPL14*-silenced plants had a significantly decreased sclerenchyma wall thickness and total surface of xylem (Figure 5D,E). The *PlSPL14*-silenced plants had a significantly increased number of xylem cells (Figure 5F), while the size of xylem cells was smaller (Figure 5G,H). These results demonstrated that the silencing of *PlSPL14* led to a reduction in stem strength, hindered stem growth and development, and slowed down stem diameter growth. Furthermore, we introduced a tobacco heterologous transformation system to study the function of *PlSPL14*. The *PlSPL14* transgenic tobacco plants had a significantly increased plant height (Figure 6D) and stem strength (Figure 6F). Micro-observation showed an increased xylem thickness and number of cell layers (Figure 7B,C). When compared with the WT plants, the *PlSPL14*-overexpressing lines had an increased sclerenchyma wall thickness and total surface of xylem (Figure 7D,E). The *PlSPL14*-overexpressing lines had a significantly increased number of xylem cells (Figure 7F). Consequently, the total surface of xylem is positively correlated with secondary xylem activity, along with the presence of sclerenchyma wall thickness, both crucial factors influencing stem strength. These results indicated that the overexpression of *PlSPL14* led to a thickening of xylem cells and increased stem strength, thereby positively regulating xylem development in *P. lactiflora*. It was initially speculated that *PlSPL14* plays a positive role in the xylem development of *P. lactiflora* based on the above results.

SPL transcription factors can also function by interacting with proteins. In *T. aestivum*, *TaPIL1* (PHYTOCHROME-INTERACTING FACTOR-LIKE) physically interacts with *TaSPL3/17* and plays an important role in inhibiting plant tillering/branching [43]. In addition, recent studies have reported the physical interaction between BZR1 and SPL9 to cooperatively regulate the expression of downstream genes and play an important role in promoting vegetative phase transition in *A. thaliana* [44]. It is hypothesized that PlSPL14 may interact with other proteins to act together on downstream target genes. To further investigate the physical mechanism by which PlSPL14 regulates stem development, the present study employed a yeast two-hybrid assay to screen the interacting protein, PlSLR1 (Figure 8A). Slender Rice 1 (SLR1), a GA-negative regulator, regulates plant height and tiller number by degrading GAs in rice [45]. This explains the significant changes in plant height in the overexpression and silencing of *PlSPL14* plant phenotypes, and it is hypothesized that an interaction between PlSLR1 and PlSPL14 forms a protein complex that cooperatively regulates downstream target genes. However, the specific regulatory mechanism is still unclear and will be verified in future research. The temporal expression analyses showed that the expression level of *PlSLR1* gradually decreased with stem development (Figure 8B), which is exactly opposite to *PlSPL14*. In future research, clarifying the precise function of *PlSLR1* will be crucial to further unravel the regulatory mechanism of genes on the GA pathway mediated by *PlSPL14*.

## 4. Materials and Methods

### 4.1. Plant Materials

The experimental materials of this study included *Paeonia lactiflora* cultivated in an open field at the National Germplasm Repository of Yangzhou University (32°23′ N, 119°24′ E). Stem segments of a 12 cm length from the top part of *P. lactiflora* were collected at four developmental stages: flower-bud stage, pigmented stage, unfolded-petal stage, and full-bloom stage (designated as S1, S2, S3, and S4, respectively). Additionally, various tissues including roots, stems, leaves and petals, vascular bundles, epidermis, and pith were harvested from plants at the S4 stage for gene cloning and expression analysis. Three-year-old *P. lactiflora* ‘Da Fugui’ was utilized for the virus-induced gene silencing (VIGS) assay. Upon collection, all samples were immediately frozen in liquid nitrogen and stored at −80 °C for subsequent use. *N. tabacum* was used for the Genetic Transformation Test (GTT) and *N. benthamiana* was used for subcellular localization, and they were both grown in a greenhouse.

### 4.2. RNA Extraction and Quantitative RT-PCR (qRT-PCR)

Total RNA were extracted from different plant samples of tobacco and *P. lactiflora* using the MiniBEST Plant RNA Extraction Kit (TaKaRa, Shiga, Japan), referring to the respective manufacturer’s instructions. To detect the concentration of RNA, a spectrophotometer (YOMIM, Hangzhou, China) was used. The first-strand cDNA was synthesized using the HiScript^®^ III 1st Strand cDNA Synthesis Kit (Vazyme, Nanjing, China), and stored at −20 °C for subsequent experiments. The qRT-PCR analysis was conducted using the NovoStart^®^ SYBR qPCR SuperMix Plus kit (NovoProtein, Shanghai, China) and Bio-Rad CFX96 (Bio-Rad, Hercules, CA, USA). The internal reference gene of tobacco and *P. lactiflora* was *γ-Tubulin* [46] and *N. tabacum Actin* (AB158612). The results were used to calculate the relative expression of candidate genes using the 2^−ΔΔCt^ method. All primers used are listed in Table S2.

### 4.3. Cloning and Bioinformatics Analysis of PlSPL14

Based on the reference sequences in the transcriptome [35], specific primers (Table S2) were designed by Primer 5.0 software. Through the reverse transcription polymerase chain reaction (RT-PCR), the CDS of *PlSPL14* and *PlSLR1* was amplified. The amplified sequences were provided in Table S3. The full-length cloning of the gene was performed by RACE technology, and primers were designed to amplify the 3′ and 5′ sequences of the *PlSPL14* (Table S2) according to the reference sequences, and 5′/3′ RACE amplification was performed according to the manufacturer’s instructions, respectively, using the SMARTer RACE 5′/3′ Kit (TAKARA, Shiga, Japan). Multiple sequence comparisons of PlSPL14 from *P. lactiflora*, and SPL proteins from *A. thaliana* and *O. sativa* were carried out using MEGA7.0 built-in ClustalW software (Table S1), and a phylogenetic tree was constructed using the neighbor-joining (NJ) method. Physicochemical properties of SPL proteins were analyzed via the ProtParam website (https://web.expasy.org/protparam/, accessed on 1 September 2023). The conserved motifs of the SPL proteins were analyzed by the MEME website (http://meme-suite.org/tools/meme/, accessed on 10 September 2023), with a maximum motif output value of 10. TBtools was used to visualize the gene structure of *SPLs* from different plants.

### 4.4. Subcellular Localization

For subcellular localization, the CDS of *PlSPL14* was amplified from cloned plasmids with specific primers, and were fused into the pCAMBIA2300 vector with eGFP at SalI restriction site by recombinant technology. The Agrobacterium strain GV3101 was subsequently transformed by the fusion vector, empty vector and mCherry protein, which was injected into *N. benthamiana* leaves. After 36 h of darkness, fluorescence images of the excitation spectra were observed by ultra-high resolution laser confocal microscopy (TCS SP8 STED, Leica, Wetzlar, Germany) in the 476 nm, 488 nm and 561 nm channels, respectively. The Green Fluorescent Protein (GFP) and the Red Fluorescent Protein (RFP) are a pair of marker proteins that are often used, and they can be observed in cells with different fluorescence microscopy, green represents auto-fluorescence localization of genes, red represents nuclear localization marker. The gene-specific primers were shown in Table S2.

### 4.5. Virus-Induced Gene Silencing (VIGS) of PlSPL14 in P. lactiflora

Based on a tobacco rattle virus (TRV)-based VIGS technology, the TRV1 and TRV2 vectors were used for silencing *PlSPL14* in *P. lactiflora* [47]. For VIGS assay, we constructed specific gene fragments into vectors to realize the silencing. In this study, we inserted a *PlSPL14*-specific fragment into the tobacco rattle virus TRV2 vector. As shown in the Figure S4, our selected *PlSPL14* sequence exhibits low similarity with other sequences, confirming specific silencing of *PlSPL14* rather than other homologous genes. The specific fragment of *PlSPL14* was amplified by specific primers and constructed into the vector Xbal and KpnI restriction sites of TRV2 using recombinant technology. Then, TRV2 vector, constructed TRV2-*PlSPL14* plasmid and TRV1 vector were transformed into Agrobacterium strain GV3101 according to the manufacturer’s instructions. The bacterial fluids of TRV2 and TRV1 were mixed in the ratio of 1:1, and the mixture was infested by vacuum suction filtration into *P. lactiflora* cultivar ‘Da Fugui’. After 30–35 days of incubation, the treated plants were firstly subjected to phenotypic observation, determination of plant height, stem thickness and stem strength. Additionally, a universal NK-2 digital force measuring apparatus (Huier, Hangzhou, China) was employed to assess stem strength. The measurement was determined by the force required to rupture the middle part of the upper stem through axial pulling. The middle parts of the upper stem were then fixed in formaldehyde-acetic acid (FAA) solution (5% formaldehyde, 5% glacial acetic acid, 63% ethanol) for microscopy observation. The remaining stems were immediately frozen in liquid nitrogen and stored at −80 °C to facilitate subsequent gene expression analysis and PCR, qRT-PCR identification. Xylem thickness and cell layer number, total surface of the xylem, sizes of xylem cells, number of xylem cells, and sclerenchyma wall thickness were measured using ImageJ. All the primers used are listed in Table S2.

### 4.6. Overexpressing PlSPL14 in Tobacco

The CDS of *PlSPL14* was amplified using a specific primer containing BamH I/Kpn I restriction sites, and constructed into the vector pCAMBIA1301 using recombinant technology. The constructed vector plasmid was introduced into the Agrobacterium tumefaciens strain GV3101 (Shanghai Weidi Biotechnology, Shanghai, China) by freeze–thawing, and then the strain was infected into the WT *N. tabacum* using a leaf disc transformation method. The WT plants and transgenic tobacco line were firstly subjected to phenotypic observation, and the determination of plant height, stem thickness, and stem strength. The middle parts of the upper stem were then fixed in FAA for microscopy observation. The remaining stems were immediately frozen in liquid nitrogen and stored at −80 °C to facilitate subsequent gene expression analysis, PCR, and qRT-PCR identification. Xylem thickness and cell layer number, the total surface of the xylem, sizes of xylem cells, the number of xylem cells, and sclerenchyma wall thickness were measured using ImageJ. All of the primers used are listed in Supplementary Data Table S2.

### 4.7. Yeast Two-Hybrid Screening

To identify the protein interacting with *PlSPL14* in the stem of *P. lactiflora*, we used the yeast two-hybrid library screening system. The CDS of *PlSPL14* was inserted into pGBKT7 (BD7) containing the Nde I restriction site to construct a BD7-*PlSPL14* bait plasmid. Firstly, self-activation and toxicity testing of the bait gene BD-*PlSPL14* was performed. Then, the BD-*PlSPL14* plasmid and the *P. lactiflora* double hybrid library plasmid pGADT7 (AD) were integrated into the Y2H yeast strain through co transformation. The transformants were screened by selective medium SD quadruple-dropout (QDO, SD/-Trp-Leu-His-Ade) with X-α-gal. After 3–5 days of cultivation at 30 °C, single colonies that turned blue on the medium were picked and homology was performed after obtaining the sequence using sangon sequencing. Furthermore, screening was performed for candidate interaction proteins for yeast two-hybrid one-to-one validation to identify the interaction protein for PlSPL14. The list of candidate interaction proteins from the yeast library screening are listed in Supplementary Data Table S4.

### 4.8. Microscopy Observation and Cell Counting

The stem segments were fixed by FAA and decolorized with an ethanol gradient series (75:25, 50:50, and 25:75; *v*/*v*). The stems were then incubated in an ethanol:xylene solution (75:25, 50:50, and 25:75; *v*/*v*). The stem segments were immersed in a 25:25 (*v*/*v*) xylene:paraffin solution overnight at 42 °C and embedded in 100% paraffin. The embedded stem segments were meticulously sectioned into 8-μm slices employing a rotary microtome (RM2235, Leica, Wetzlar, Germany). Subsequently, the sections underwent deparaffinization using 100% xylene followed by a solution of ethanol:xylene (50:50; *v*/*v*), and were gradually hydrated through a series of ethanol solutions. Following this preparation, the sections were stained with a solution containing 5% (*w*/*v*) tolonium chloride, and were observed under the lenses of two optical microscopes (SZX7 and CX31RTSF, Olympus, Tokyo, Japan). Tolonium chloride is a polychromatic alkaline dye that stains different components of plant tissues and cells. The lignified part is blue-green, the phloem is blue-purple, and other parts are a lighter blue-green.

### 4.9. Statistic Analysis

All data and measurements were conducted at least in triplicate, and the error bars represent the standard deviation. Different letters indicate the significant differences (*p* < 0.05) based on a Student’s *t*-test, and GraphPad Prism 9 was used for charts.

## Data Availability

Data are contained within the article and Supplementary Materials.

## Supplementary Materials

The following supporting information can be downloaded at: https://www.mdpi.com/article/10.3390/ijms25158443/s1.
