# Supplementary material for: Identification and Functional Studies on the Role of PlSPL14 in Herbaceous Peony Stem Development
Source: Int J Mol Sci. 2024 Aug 2;25(15):8443. doi: 10.3390/ijms25158443 (PMC11313244; doi:10.3390/ijms25158443)
Supplement: Supplementary file 1 [file ijms-25-08443-s001.zip › Supplementary Figures.pdf]

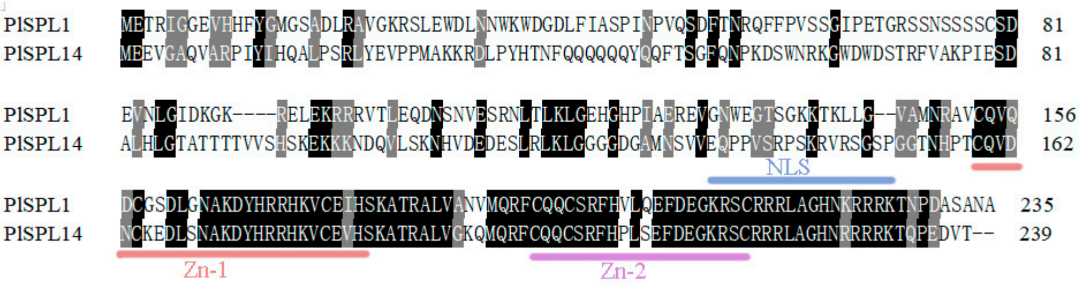

Figure S1. Key domain analysis of PlSPL1 and PlSPL14.

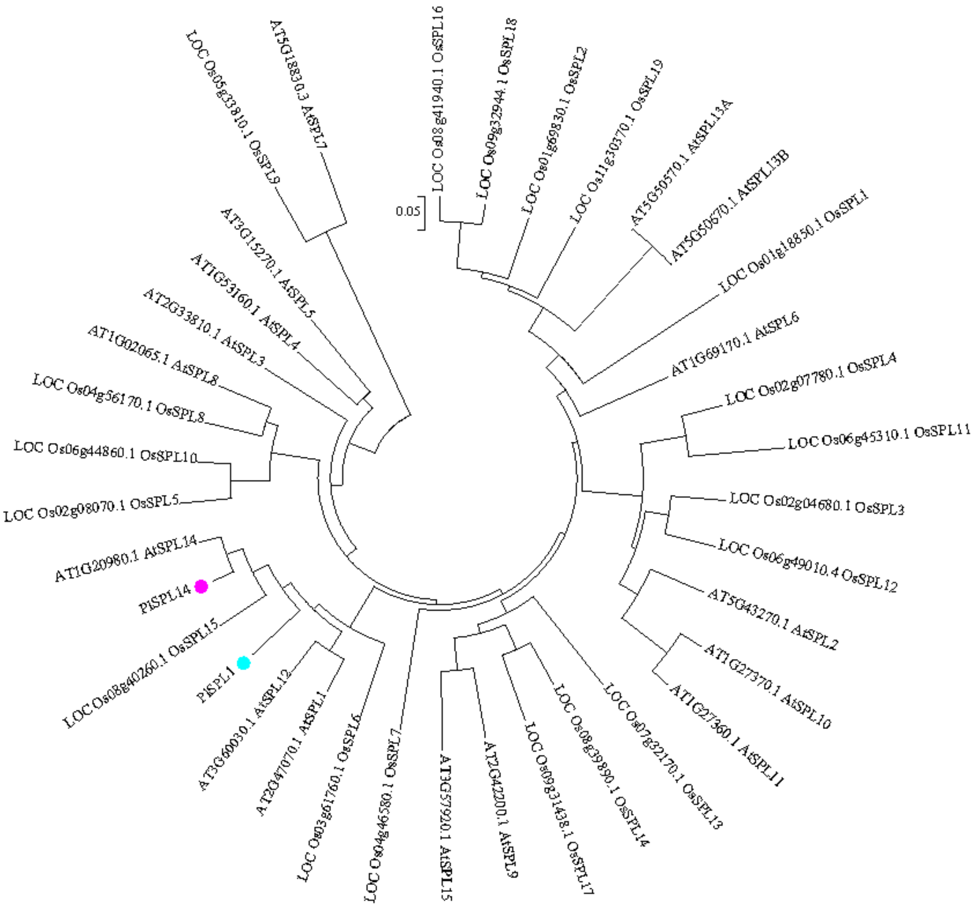

Figure S2. Phylogenetic analysis of SPL proteins from herbaceous peony (*P. lactiflora*), arabidopsis (*A. thaliana*) and rice (*O. sativa*).

	Number of amino acids	Molecular formula	Molecular weight	Theoretical pI	Total number of atoms	Instability index
PlSPL1	999	$C_{4869}H_{7682}N_{1406}O_{1497}S_{43}$	111.25	6.26	15,497	47.54
PlSPL14	1085	$C_{5208}H_{8288}N_{1538}O_{1629}S_{42}$	119.86	8.75	16,705	52.50

Figure S3. Comparative analysis of protein physicochemical properties between PlSPL1 and PlSPL14.

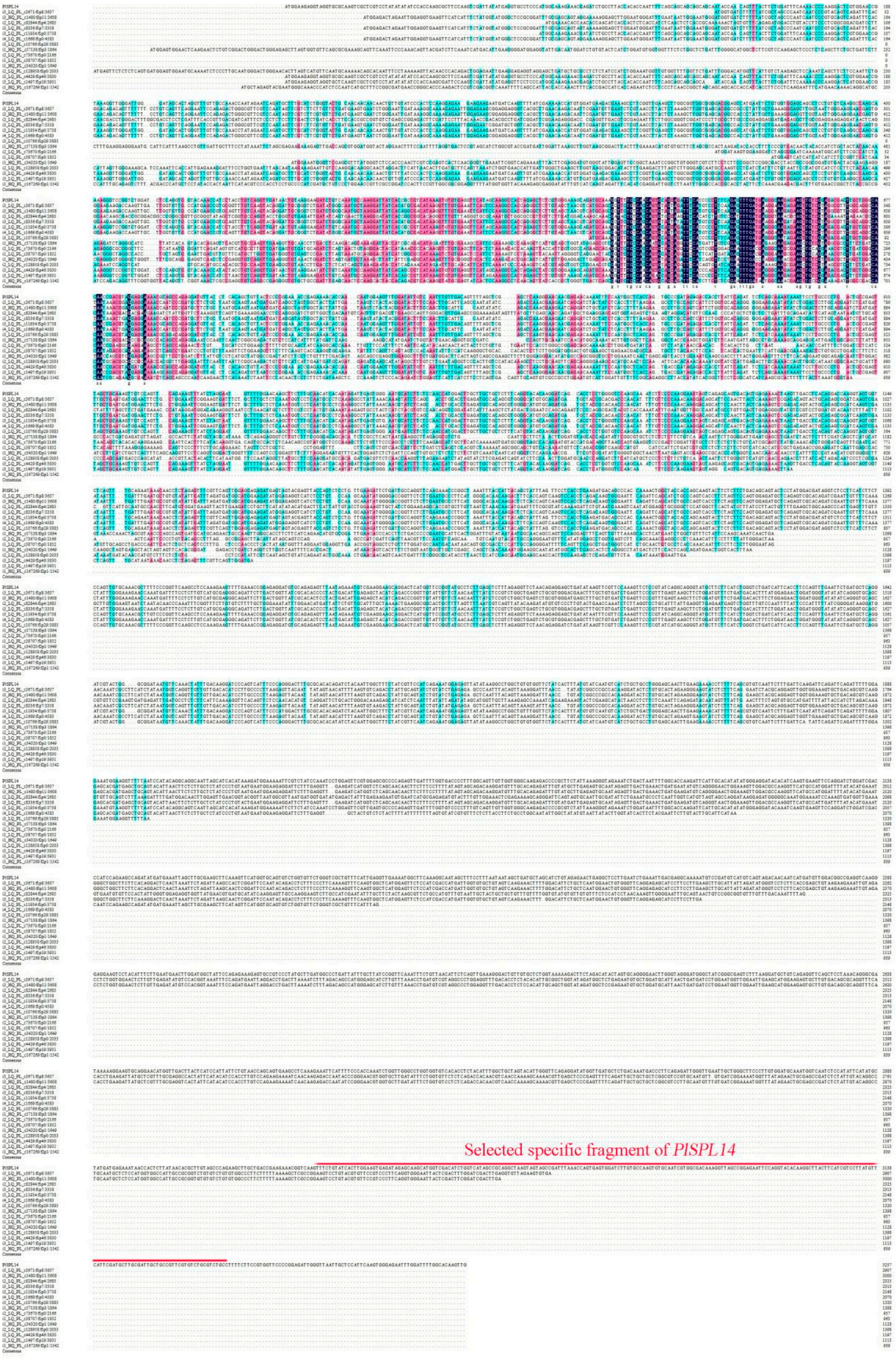

Figure S4. Comparative nucleotide sequence analysis of *PlSPL14* and its homologous genes.
